# Nationwide Incidence of Chigger Mite Populations and Molecular Detection of *Orientia tsutsugamushi* in the Republic of Korea, 2020

**DOI:** 10.3390/microorganisms9081563

**Published:** 2021-07-22

**Authors:** Min-Goo Seo, Bong-Goo Song, Tae-Kyu Kim, Byung-Eon Noh, Hak Seon Lee, Wook-Gyo Lee, Hee Il Lee

**Affiliations:** Division of Vectors and Parasitic Diseases, Korea Disease Control and Prevention Agency, 187 Osongsaenmyeong2-ro, Osong-eup, Heungdeok-gu, Cheongju 28159, Korea; koreasmg@korea.kr (M.-G.S.); gign1204@korea.kr (B.-G.S.); tkkim80@korea.kr (T.-K.K.); nbudia@korea.kr (B.-E.N.); hslee8510@korea.kr (H.S.L.); twinleo@korea.kr (W.-G.L.)

**Keywords:** chigger, mite, *Orientia tsutsugamushi*, rodent, scrub typhus, tsutsugamushi disease

## Abstract

The Korea Disease Control and Prevention Agency has established regional centers at 16 locations to monitor vectors and pathogens. We investigated the geographical and temporal distribution of chigger mite populations to understand tsutsugamushi disease epidemiology in 2020. To monitor weekly chigger mite populations, 3637 chigger mites were collected from sticky chigger mite traps in autumn. Chigger mites appeared from the first week of October to the third week of December, peaking in the fourth week of October. The predominant species were *Leptotrombidium scutellare*, *Leptotrombidium palpale*, *Neotrombicula kwangneungensis*, *Neotrombicula tamiyai*, and *Leptotrombidium pallidum*. To monitor *Orientia tsutsugamushi* infection in chigger mites, 50,153 chigger mites were collected from 499 trapped wild rodents in spring and autumn, with a chigger index of 100.5. Approximately 50% of chigger mites were pooled into 998 pools, and the minimum infection rate (MIR) of *O. tsutsugamushi* was 0.1%. Jeongeup had the highest MIR for *O. tsutsugamushi* (0.7%). The Kato-related genotype was the most common (52.2%), followed by the Karp-related (17.4%), Boryong (13.0%), JG-related (8.7%), Shimokoshi (4.3%), and Kawasaki (4.3%) genotypes. Ecological and geographical studies focusing on the basic ecology and pathology of mites will improve our understanding of tsutsugamushi disease risks in the Republic of Korea.

## 1. Introduction

Scrub typhus, also known as tsutsugamushi disease, is caused by one of the world’s oldest known vector-borne pathogens, *Orientia tsutsugamushi*. This species is an obligate intracellular Gram-negative bacterium that requires host cells and is transmitted to humans by bites from the larvae of chigger mites [1]. This disease is a severe public health problem in the Asia-Pacific region, including Japan, the Republic of Korea, China, Thailand, Taiwan, Indonesia, the Philippines, and India [2]. In 2020, 4457 patients were reported to have tsutsugamushi disease, and 2719 (61.0%) cases occurred in the southern areas of the Republic of Korea [3]. Generally, tsutsugamushi disease is reported in late September and peaks in November in the Republic of Korea [3]. The essential feature of this disease is that it is arthropod-mediated, maintaining the infection in nature through the association among warm-blooded animals, such as humans and small wild mammals, such as mites, with the pathogen *O. tsutsugamushi* [4].

Trombiculidae is a family of nationwide-distributed mites in the Republic of Korea, with 15 genera and 63 species reported in the country. Among them, eight species, including *Euschoengastia koreaensis*, *Helenicula miyagawai*, *Leptotrombidium scutellare*, *Leptotrombidium palpale*, *Leptotrombidium pallidum*, *Leptotrombidium orientale*, *Leptotrombidium zetum*, and *Neotrombicula japonica*, can carry *O. tsutsugamushi* [5]. Trombiculidae mites go through a four-stage life cycle (eggs, larvae, nymphs, and adults). The larvae of the mites (called chiggers) only feed on the body fluids of animal hosts, whereas nymphs and adults feed on insect eggs in the ground or under leaves [6].

Traditional surveillance methods for chigger mites include trapping rodents and harvesting engorged chigger mites. This technique has merits, such as the large-scale collection of engorged larvae [7,8,9,10]; however, engorged chigger mites do not provide information on the tsutsugamushi disease epidemic because they do not feed again and cannot transmit *O. tsutsugamushi* to humans [11]. In addition, it is difficult to use this monitoring method for weekly data collection because frequent capture might affect rodent ecology. Information on chigger mites can be essential for a comprehensive tsutsugamushi disease epidemiology program, including bionomics, larval habitats, population density, and species distribution [7]. However, the use of only chigger mite prevalence information and infection rates of chigger mites is not sufficient to explain the tsutsugamushi disease epidemic, as other ecological and geographical factors must be considered.

The prevalence of *O. tsutsugamushi* in chigger mites is a critical tool for studying the epidemiology of chigger mites and chigger mite-borne pathogens. The significance of changing habitats, climate, and environments on the risk of infectious zoonotic diseases is becoming increasingly recognized [12]. For example, climate change is associated with the northern expansion of the key vector *L. scutellare* [13]. Thus, understanding the role of chigger mites as vectors of *O. tsutsugamushi* and the proportion of infected individuals is crucial for appropriate chigger mite control programs, particularly considering global warming scenarios. Since 2010, the Korea Disease Control and Prevention Agency has established regional centers to monitor the population density and pathogens associated with climate change [14]. In this study, we collected unfed chigger mites to monitor the geographical and temporal distribution and weekly fluctuations in chigger mite populations. We also collected engorged chigger mites from rodents in the spring and autumn seasons to monitor the risk factors associated with the relationship between the genetic characteristics and occurrence of *O. tsutsugamushi*. These results could be used to estimate the source of infections in upcoming epidemiological research and provide risk assessments of vector-borne pathogens based on the effects of global warming.

## 2. Materials and Methods

### 2.1. Ethical Approval

The animal protocol used in this study was reviewed and approved according to the guidelines for ethical procedures and scientific care by the Institutional Animal Care and Use Committee of the Korea Centers for Disease Control and Prevention (KCDC-093-18). There was no need for specific permission for each collection site because they were not located within national parks or protected areas. The collected wild rodents were not endangered or protected species in the Republic of Korea.

### 2.2. Weekly Incidence of Chigger Mite Populations

Chigger mites were collected using sticky chigger mite traps (190 × 240 × 150 mm; Patent No. 10-1555975) at 16 collection sites nationwide in 2020. Each collection site consisted of four different environmental points: a rice paddy field, dry paddy field, waterway, and meadow. At each point, we installed five traps (a total of 20 traps per collection site) and collected 16 weekly samples from the 36th week of the year (4th week of August) to the 51st week of the year (3rd week of December).

The trap consisted of two primary parts, a steel body and a top lid. The steel body served as a collection spot for climbing chigger mites and contained an attractant. The attractant (Attractants, E-TND, Republic of Korea) was a lure located in the upper meshed part of the steel body that mimics the human skin odor and contains a sliding side for the sticky tape. The top lid protected the steel body from rain and sunlight and contained the sticky tape. This collection method was based on the principle that the lure attracted questing chigger mites. They would crawl up the steel body and then be captured by the sticky tape. The sticky tapes were brought back weekly to the laboratory. The collected chigger mites were examined through microscopy, detached from the sampling tape using lamp oil, and stored in 70% ethanol for identification.

### 2.3. Identification of Chigger Mites

To recover the chigger mites from the sticky chigger mite trap, individual chigger mites were transferred to glass slides and mounted with polyvinyl alcohol mounting medium (BioQuip, CA, USA). The samples were identified at the species level by optical microscopic examination using morphological keys [15].

### 2.4. Surveillance of O. tsutsugamushi in Chigger Mites from Wild Rodents

Chigger mites were collected from wild rodents captured at 16 collection sites nationwide in 2020 using rodent traps. Each collection site consisted of five environmental points: rice paddy fields, dry paddy fields, reservoirs, waterways, and hillsides, which are very common types of rural area in the Republic of Korea. The collections were performed at each location four times in the spring (4th week of March, and 2nd week of April) and autumn (4th week of October, and 2nd week of November).

In total, 100 Sherman live folding traps (3 × 3 × 9 inches, BioQuip, CA, USA) baited with peanut butter-spread biscuits were set up at each point in the collection sites at 3–5 m intervals (20 traps per environmental point) and collected the following day. The collected wild rodents were transported to the laboratory and identified using taxonomic keys [13]. After being euthanized via compressed carbon dioxide (CO_2_), the wild rodent corpses were hung over glass bowls filled with tap water for 24 h to harvest chigger mites. The chigger mites were recovered from the water surface with a fine brush and stored at 4 °C for further study.

### 2.5. Molecular Detection of O. tsutsugamushi from Chigger Mites

To recover the chigger mites from wild rodents, approximately half of them were used to detect *O. tsutsugamushi* infection, and the remaining chigger mites were preserved for long-term use as a biological resource. The chigger mite pools (1–30 mites per rodent) were homogenized using Precellys^®^ CK28-R Lysing kit (bead tube for hard tissue homogenization, Bertin Technologies, Bretonneus, France) and Precellys^®^ evolution (Homogenizer, Bertin Technologies), followed by genomic DNA extraction from the pools using a commercial G-spin^TM^ total DNA extraction kit (iNtRON Biotechnology, Korea) according to the manufacturer’s instructions. The pathogen *O. tsutsugamushi* was detected using a LiliF^TM^ TSUTSU Nested PCR kit (iNtRON Biotechnology, Seoul, Korea) with DNA samples via nested PCR according to the manufacturer’s protocol. Species-specific primers used were specific for the protein antigen gene 56 kDa of *O. tsutsugamushi*. The minimum infection rates (MIRs, no. positive pool of mites/total no. mites tested × 100) were subsequently calculated.

### 2.6. DNA Sequencing and Phylogenetic Analysis

The purified PCR products were obtained using forward and reverse PCR amplification primers and then sequenced by Macrogen (Seoul, Korea). Each raw chromatogram was visually inspected to detect double peaks using the CLC Main Workbench 6.9 (CLC Bio, Qiagen, Aarhus, Denmark). Sequences were analyzed using the multiple sequence alignment program CLUSTAL Omega (version 1.2.1). The results of sequence alignments were modified using BioEdit (version 7.2.5) and analyzed using a similarity matrix. A phylogenetic analysis was performed with MEGA (version 6.0) using the maximum likelihood method and assessed using bootstrap analysis with 1000 replicates.

### 2.7. Geographical and Climate Analyses

Distribution maps were drawn by interpolation using the inverse distance weighted (IDW) technique among spatial analyst tools in ArcGIS 9.0 (2004, Environmental Research Systems Institute, Redlands, CA, USA) to compare the geographical distribution of chigger mites. We used the 2020 regional mean temperature climate data measured by the Korea Meteorological Administration in the Republic of Korea.

## 3. Results

### 3.1. Weekly Prevalence of Chigger Mite Populations

**Figure 1:**
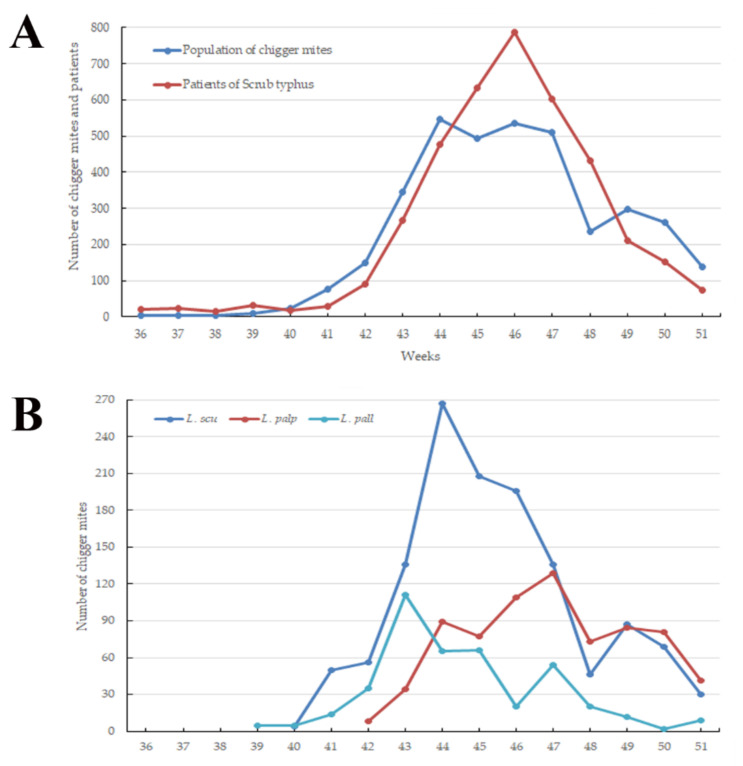
Weekly fluctuations in the population of chigger mites and patients of tsutsugamushi disease (**A**), and the abundance of the three predominant chigger mite species carrying *Orientia tsutsugamushi* (**B**) collected from the sticky traps in the Republic of Korea between the 36th and 51st weeks in 2020. *L. scu, Leptotrombidium scutellare; L. palp, Leptotrombidium palpale; L. pall, Leptotrombidium pallidum*.

In total, 3637 chigger mites representing 5 genera and 15 species were collected from sticky chigger mite traps. Chigger mites were first reported in the Cheorwon (three mites) and Hwaseong (one mite) regions during the 36th week of the year (4th week of August). The chigger mites were collected in the Seogwipo region (one mite) during the 44th week (4th week of October) (data not shown). The main incidence of chigger mites occurred from the 41st week (1st week of October) to the 51st week (3rd week of October) of the year. The highest peak was observed at the 44th week (4th week of October; Figure 1). Among the sampled regions, the Hwaseong (16.3%, 594/3637) and Boryeong (0.4%, 16/3637) regions had the highest and lowest numbers of collected chigger mites, respectively. Among the environmental points, rice paddy fields (42.5%, 1544/3637) had the highest incidence of chigger mites among all trapping points, followed by waterways (26.0%, 947/3637), dry paddy fields (16.7%, 608/3637), and meadows (14.8%, 538/3637) (data not shown).

Among the chigger mite species collected in this study, the eight chigger mite species carrying *O. tsutsugamushi* (Table 1) included *L. scutellare* (35.3%, 1285/3637), *L. palpale* (19.9%, 725/3637), *L. pallidum* (11.5%, 418/3637), *H. miyagawai* (1.5%, 53/3637), *L. orientale* (0.8%, 29/3637), *N. japonica* (0.6%, 23/3637), *E. koreaensis* (0.2%, 7/3637), and *L. zetum* (0.03%, 1/3637).

**Figure 2:**
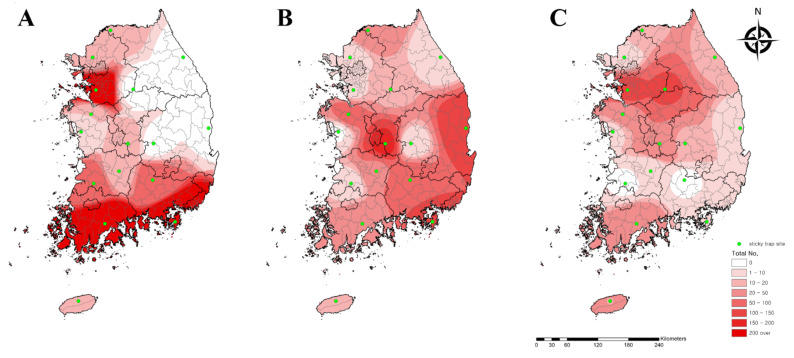
Geographical distribution of three major chigger mite species carrying *Orientia tsutsugamushi*. (**A**) *Leptotrombidium scutellare*, (**B**) *L. palpale*, and (**C**) *L. pallidum* were collected from sticky traps at 16 collection sites nationwide in the Republic of Korea in 2020. The map color indicates the total number (0 to >200). Sticky traps are denoted by green dots.

We analyzed the collected data using the IDW interpolation method in ArcGIS to assess the geographical distribution of chigger mites in the Republic of Korea. Initially, we drew distribution maps based on the number of chigger mites for the three predominant species carrying *O. tsutsugamushi* (Figure 2). *Leptotrombidium palpale* and *L. pallidum* were generally distributed nationwide, especially in the central and southeastern areas and northwestern areas of the country, respectively. *L. scutellare*, however, was primarily found in the western and southern areas.

### 3.2. Prevalence of O. tsutsugamushi in Chigger Mites from Wild Rodents

In total, 6400 traps were installed, and 499 wild rodents were captured at 16 collection sites nationwide. Among the trapped wild rodents, *Apodemus agrarius* (76.4%, 381/499) was the dominant species, followed by *Crocidura lasiura* (18.8%, 94/499), *Craseomys regulus* (2.4%, 12/499), *Micromys minutus* (1.8%, 9/499), *Apodemus peninsulae* (0.4%, 2/499), and *Microtus fortis* (0.2%, 1/499) (Table 2). Among environmental points, waterways (30.9%, 154/499) had the highest trapping rates, followed by hillsides (22.7%, 113/499), reservoirs (20.6%, 103/499), rice paddy fields (14.8%, 74/499), and dry paddy fields (11.0%, 55/499) (data not shown). From these rodents, 50,153 chigger mites were collected, with a chigger index (CI, number of chigger mites per rodent) of 100.5 (Table 2). For the rodent species, *M. fortis* (224.0) and *Crocidura* spp. (10.6) had the highest and lowest CI, respectively.

**Figure 3:**
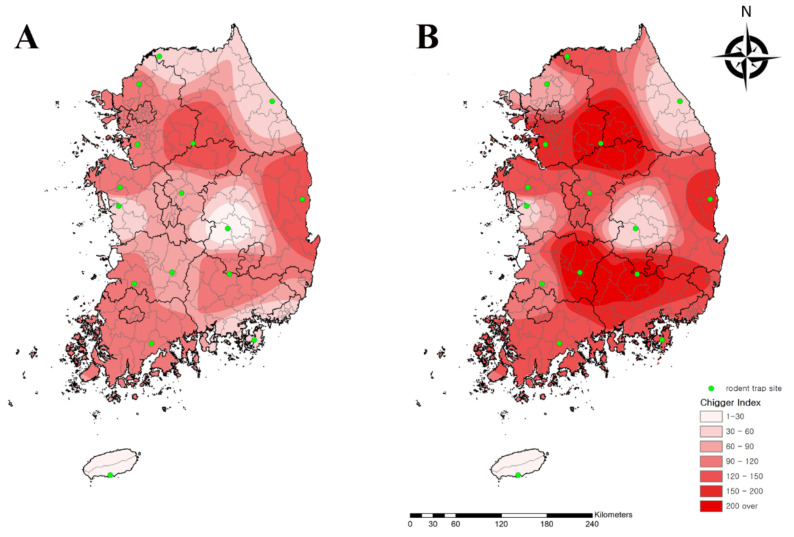
Geographical distribution of chigger mites from rodents collected from rodent traps at 16 collection sites nationwide in the (**A**) spring and (**B**) autumn in the Republic of Korea in 2020. The map color indicates the chigger index (0 to >200). Rodent traps are denoted by green dots. The chigger index shows the number of chigger mites per rodent.

The number of collected rodents were similar in the spring (257) and autumn (242) (data not shown), but the CI of chigger mites in autumn (127.8) was 1.7-fold higher than that of spring (75.1) (Figure 3). Among all regions, the Yeoju (194.8) and Seogwipo (16.9) regions had the highest and lowest CI, respectively (Table 3).

Among the 50,153 chigger mites sampled from wild rodents, 24,937 (~50%) were pooled into 998 pools, and the MIR of *O. tsutsugamushi* was 0.1% (23 pools/24,937 mites; Table 3). The chigger mites collected from the Jeongeup (0.7%) region had the highest MIR of *O. tsutsugamushi*, followed by those of Hwaseong (0.2%), Hapcheon (0.1%), Cheorwon (0.1%), Boseong (0.1%), Yeongdeok (0.1%), and Yeoju (0.03%).

### 3.3. Molecular and Phylogenetic Analyses

**Figure 4:**
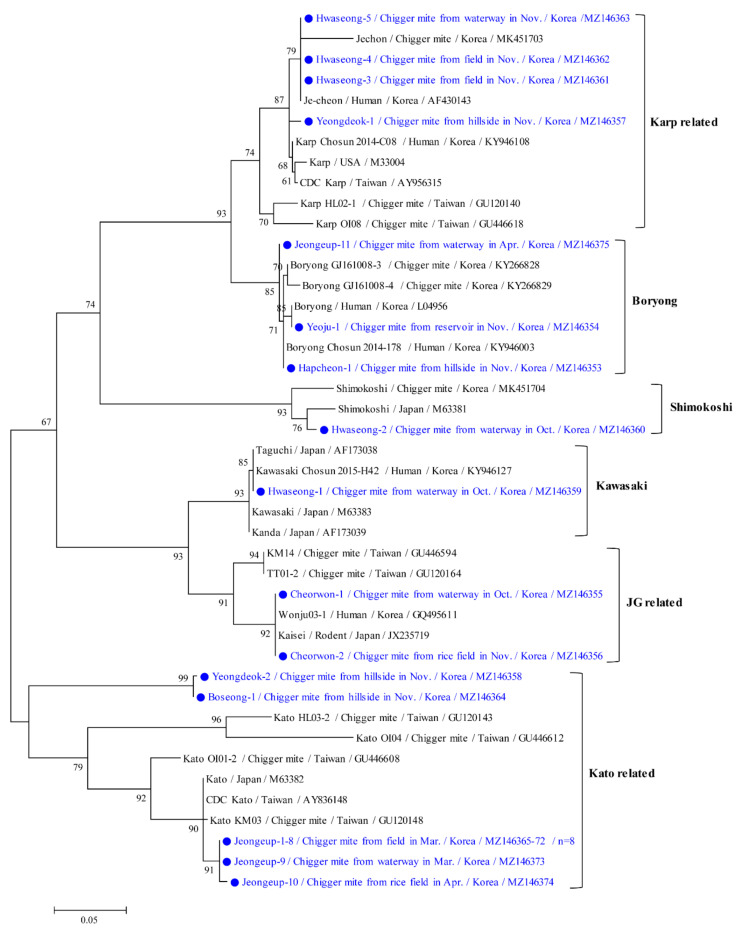
Phylogenetic tree of *Orientia tsutsugamushi* based on 56 kDa gene sequences. The maximum likelihood method was used to create the tree, and closed circles and blue letters indicate the sequences detected in this study. The GenBank accession numbers are shown, and the *O. tsutsugamushi* genotypic groups are indicated. Branch numbers indicate bootstrap support levels (1000 replicates), and the scale bar displays the substitution numbers for each nucleotide.

Phylogenetic analyses showed that the 56 kDa protein of *O. tsutsugamushi* (Figure 4) was clustered with formerly documented sequences that were divided into six groups. The Kato-related genotype (52.2%, 12/23) was the most common, followed by the Karp-related (17.4%, 4/23), Boryong (13.0%, 3/23), JG-related (8.7%, 2/23), Shimokoshi (4.3%, 1/23), and Kawasaki (4.3%, 1/23) genotypes. Furthermore, chigger mites from one *M. fortis* rodent in the Jeongeup region were only infected with the Kato genotype (five positive pools). In contrast, chigger mites from several *A. agrarius* rodents throughout the country were infected by all genotypes (18 positive pools) of *O. tsutsugamushi* (data not shown).

The twelve Kato-related strains found in this study shared 86.9–100% identity and shared 85.8–94.9% identity with previously reported Kato-related *O. tsutsugamushi* isolates from GenBank. The four Karp-related strains found in this study shared 98.45–100% identity and shared 94.7–99.1% identity with previously reported Karp-related strains. The three Boryong strains shared 98.8–99.5% identity with each other and shared 95.4–100% identity with previously reported Boryong-related strains. The representative sequences reported in the present study have been submitted to GenBank under the following accession numbers: MZ146353–MZ146375 (*O. tsutsugamushi* 56 kDa).

## 4. Discussion

Some researchers have doubted that chigger mites of non-vector species would harbor *Rickettsia* parasites for some time after they feed on infected rodents [16]. While feeding, uninfected chigger mites can acquire *O. tsutsugamushi* from infected mammalian hosts, and the acquired *O. tsutsugamushi* is transstadially transmitted to the adult stage but rarely vertically (transovarially) transmitted to the progeny [16]. However, some possibility remains for *O. tsutsugamushi* to spread in the chigger mite population from wild small mammals. The existing evidence suggests that chigger mites might act as both a reservoir and host of the disease. There is much doubt about the interaction between transstadial and transovarial *O. tsutsugamushi* transmission. The lack of evidence of vertical transmission of *O. tsutsugamushi* following acquisition from an infected host in the laboratory led to speculation that this occurrence might be so rare that it is perhaps irrelevant in nature [17]. Chigger mite co-feeding on rodents seems to be more related to the effective transmission of *O. tsutsugamushi* than feeding on rickettsemic hosts [18]. However, infection in humans occurs only through the bite of unengorged chigger mites carrying *O. tsutsugamushi*, which is transovarially received from infected female parents [19].

Another clinical and epidemiological opinion is that unengorged chigger mites are more critical and appropriate than engorged chigger mites, primarily in determining chigger vectors. Other researchers have used unfed chigger mites to assess the true reservoir of *O. tsutsugamushi* [11,13,20,21]. In nature, infected chigger mites have a high transmission ability. There were some previous studies presenting two infection rates as indicators of transovarian development; however, these rates might not have any relation to each other. One is the filial infection rate, which is the percentage of infected offspring derived from an infected female. The other is the transovarian infection rate, which is the percentage of infected females that pass rickettsiae to their offspring [22]. For example, the transovarian infection rate and filial infection rate were estimated to be 100% and 95–100% in *Leptotrombidium deliense*, 98% and 90% in *Leptotrombidium fletcheri*, 100% and 20–100% in *Leptotrombidium arenicola*, and 100% and 97% in *L. pallidum*, respectively [22].

In this study, to monitor the geographical and temporal distributions of chigger mite species, we collected unfed chigger mites from sticky traps weekly at environmental points, a more accessible approach to human activities in rural areas in the autumn season, which is associated with a high incidence of tsutsugamushi disease. Unfortunately, we could not test *O. tsutsugamushi* infections in unfed chigger mites, and further studies are needed to analyze the *O. tsutsugamushi* prevalence in unfed larvae in an epidemiologically meaningful manner. Other studies have used unfed larvae to identify the prevalence of *O. tsutsugamushi* infection, including *L. scutellare* (3.0%) and *N. japonica* (100%) in Japan [11], *H. miyagawai* (MIR 2.6%) in the Republic of Korea [13], *L. scutellare* (0.01%) in Japan [20], and *L. pallidum* (15.6%) and *L. intermedium* (0.1%) in Japan [21]. Normally, it is difficult to individually conduct both species identification and an assessment of infection rates of *O. tsutsugamushi* in large-scale assays of chigger mites. For example, among 6350 chigger mites collected from rodents, only 830 were tested for *O. tsutsugamushi* infection [23]. Thus, we also collected engorged chigger mites from rodents in the spring and autumn to determine the risk factors associated with the relationship between the genetic characteristics and occurrence of *O. tsutsugamushi*. We found that the MIR of *O. tsutsugamushi* was 0.1% in engorged chigger mites from rodents. Other studies used engorged chigger mites from rodents to identify the prevalence of *O. tsutsugamushi* infection in the Republic of Korea, such as those on chigger mites (MIR, 0.6%) [10], *L. scutellare* (0.6%), *L. palpale* (0.9%), and *L. orientale* (0.1%) [24], *L. scutellare* (0.6%) and *L. orientale* (0.4%) [25], *L. scutellare* (11.3%), *L. pallidum* (9.5), *L. palpale* (1.1%), *L. orientale* (11.4%), and *E. koreaensis* (11.8%) [26], *L. scutellare* (0.3%) and *L. orientale* (0.2%) [27], and *L. scutellare* (3.7%), *L. pallidum* (1.5), *L. palpale* (5.3%), *L. orientale* (3.6%), *N. japonica* (4.3%), and *E. koreaensis* (1.9%) [23].

Questing chigger mites from our collection traps were analyzed in weekly surveys in the autumn season, from the 36th (4th week of August) to the 51st (3rd week of December) week of 2020. We identified the geographical distribution of chigger mite species and weekly fluctuations in these insects. During the entire period, the northern areas of the Hwaseong (16.3%, 594/3637) and Cheorwon (15.1%, 550/3637) regions and the southern area of the Geoje (13.9%, 507/3637) and Boseong (11.9%, 431/3637) regions accounted for more than half of the chigger mites collected, suggesting their endemicity. Chigger mites were first collected in the Cheorwon (three *L. tectum*) and Hwaseong (one *N. tamiyai*) regions during the 36th week of 2020, whereas they were collected in the Seogwipo (one *L. scutellare*) region during the 44th week of 2020. The Cheorwon and Hwaseong regions are northern, whereas the Seogwipo region is an island in the southernmost region of the Republic of Korea. This result suggests that the first occurrence of chigger mites tends to be delayed at lower latitudes due to temperature differences at each collection region. *L. tectum* was mostly distributed in the Cheorwon (93.7%, 119/127) region, while *N. tamiyai* was mostly distributed in the Hwaseong (11.8%, 53/448), Cheorwon (43.1%, 193/448), and Jeongeup (southern areas of the Republic of Korea, 24.1%, 108/448) regions.

The high incidence of tsutsugamushi disease from October to December is generally due to the high chigger mite population that peaks from September to November and suddenly decreases and persists at low densities from December to August [7]. An increased density of chigger mite populations increases the chances of biting humans, offering some evidence for the high seasonal incidence rates [7]. This study assessed the correlation between the emergence of chigger mites and the incidence of patients, analyzed at 2-week intervals from the first collection date of chigger mites to the first outbreak in patients. This interval corresponded to the incubation time of 7 and 10 days for tsutsugamushi disease [28]. Among chigger mite species, the temporal distribution of *L. scutellare,* which was the most prevalent species in this study, and the incidence of patients correlated with the 2-week interval. In addition, the geographical distributions of *L. scutellare* correlated more with the endemic region of patients than with other species of chigger mites (data not shown). Although further studies are needed to identify *O. tsutsugamushi* infections between chigger mites and humans, *L. scutellare* appears to be an effective vector for human transmission in the Republic of Korea.

The primary chigger mite incidence varied from the 41st to the 51st week of the year, and the highest peak in the population was observed during the 44th week of 2020. In total, 139 chigger mites were collected from all regions of the Republic of Korea in early winter at the 51st week of 2020, when the average temperature was −3.3 °C. The proportion of patients with *O. tsutsugamushi* infection in December 2020 was 10.1% (449/4457). Thus, further studies are needed to analyze the population of chigger mites and patients in autumn and winter. Continuous monitoring is required to assess the patterns of chigger mites and patient infections.

Among the three major species of chigger mites related to *O.* *tsutsugamushi,* the highest incidence periods differed by species. *Leptotrombidium scutellare* had its highest peak during the 44th week, whereas *L. palpale* peaked during the 47th week of 2020. The incidence of *L. pallidum* showed its highest peak during the 43rd week of the year. Among these species, *L. scutellare* (35.3%) and *L. palpale* (19.9%) were the most predominant in this study. However, in other studies, different proportions of chigger mite species were reported in other regions and seasons in the Republic of Korea, including the following: (1) *L.* *pallidum* (52.6%), *L. scutellare* (27.1%), and *L. palpale* (8.2%) during spring and autumn between 2005 and 2007 throughout the country [9]; (2) *L. pallidum* (43.5%), *L. orientale* (18.9%), and *L. scutellare* (18.1%) over the entire year between 2006 and 2007 in the eastern and southern areas [10]; (3) *L.* *pallidum* (74.9%), *L. scutellare* (18.9%), and *L. palpale* (2.7%) from October to November in 2006 throughout the country [7]; (4) *L. scutellare* (44.4%), *L. orientale* (22.7%), and *L.* *pallidum* (15.8%) during the entire year between 2014 and 2018 in the southwestern region of the Republic of Korea [24]; (5) *L.* *pallidum* (53.9%), *L. orientale* (13.2%), and *L. scutellare* (9.5%) in April and November in 2019 throughout the country [5]. In this study, we identified only chigger mite species from sticky traps during autumn, and the incidence of *L. palpale* (19.9%) was higher than that of *L.* *pallidum* (11.5%). This result is similar to that of other studies where the density of *L. palpale* and *L. scutellare* increased in the late autumn to winter, whereas that of *L.* *pallidum* decreased more in autumn than in spring [9,10]. Such population density variances might be attributed to the endemicity of the collection areas and other unaccounted environmental aspects.

In this study, the distribution pattern of *L. scutellare* was determined to be in the southern area (54%), where tsutsugamushi disease is prevalent, and this species is also primarily distributed in the southern and western regions of the Republic of Korea [7,9]. In a previous 1996 study, *L. scutellare* was only found to be distributed in the southern region of the country, where the annual mean temperature is >10 °C [29]. The other study reported that *L. scutellare* was primarily distributed in the southern and western areas of the Republic of Korea between 2005 and 2007 [9]. In 2015, *L. scutellare* was reported in the northwestern region of the country [4]. Here, we also found *L. scutellare* in the northwestern regions of the Republic of Korea in 2020. This result suggests that the distribution of vectors and incidence of tsutsugamushi disease have increased in the northern areas compared to the past, and this might be related to global warming [13]. Furthermore, although tsutsugamushi disease seems to be a rural disease, urban cases have been reported, particularly the metropolitan capital city of Seoul, located in the northwestern areas of the Republic of Korea [13]. The density of chigger mite species varies according to the region, season, and year. Thus, further studies are needed to analyze the distribution of chigger mites and understand the epidemiology of tsutsugamushi disease and its risk to public health in the Republic of Korea.

In this study, chigger mites were more prevalent in the autumn than in the spring. In the Republic of Korea, adult mites lay eggs in the summer [28]. The population density of chigger mite larvae was low in the summer and increased in September. Therefore, this result indicates that the high density of chigger mites might affect the high incidence rate (80.0%) of tsutsugamushi disease in the autumn. However, the difference in the number and CI of chigger mites between spring and autumn does not explain the difference in tsutsugamushi disease incidence in the same seasons [9]. In the Republic of Korea, spring is the sole seeding season, whereas autumn is the main harvest season. This could be explained by the seasonal differences in human agricultural activity patterns, suggesting that few weeds or grasses shelter chigger mites in the spring, whereas the grass and crop species richness in autumn increases the chance of contact between humans and vectors, such as chigger mites, when the vector population densities are high [10].

The fed chigger mites from rodents were analyzed regionally in March, April, October, and November. We performed the molecular detection and phylogenetic analysis of *O. tsutsugamushi* in chigger mites. Although we expected that highly endemic regions would show a higher chigger mite population and detection rate of *O. tsutsugamushi* in chigger mites, we were unable to find any relationship among the relative rates of tsutsugamushi disease, unfed chigger mite populations, CI, and *O. tsutsugamushi* infection in chigger mites. For example, the Geoje region, which had the highest number of reported cases in 2020 (*n* = 138), had a relatively low CI (74.3) compared with the highest CI (194.8) in the Yeoju region, where there were no reported cases in 2020. In addition, the Yeongdeok region reported one infected patient with *O. tsutsugamushi* but had a relatively high CI (148.9), and the Jinan region reported a relatively low number of infected patients (*n* = 19) with *O. tsutsugamushi* but had a relatively high CI (150.6). Between *O. tsutsugamushi* infection in chigger mites and the number of reported patients, we were not able to find a relationship. For example, the Geoje region (138 patients) had no MIR, the Yeongdeok region (one patient) had a relatively high MIR (0.13%), and the Hwaseong region (21 patients) had the second highest MIR (0.2%). An exception was the Jeongeup region, which had the second highest number of reported cases in 2020 (*n* = 51) while having the highest MIR (0.7%). Meanwhile, unfed chigger mite populations from sticky traps at environmental points served as a more accessible approach to human activities in rural areas. They tended to have a stronger correlation with the number of reported patients. The Geoje and Boseong regions had a high number of patients (*n* = 138 and 41, respectively) and high incidence (13.9% and 11.9%, respectively), while the Yeoju region had no patients and low incidence (3.4%). An exception was the Cheorwon region, which had a low number of reported cases (*n* = 1) but the second highest incidence (15.1%) of chigger mites. These results suggest that chigger mite populations and infection rates of chigger mites alone do not explain the tsutsugamushi disease epidemicity. Other sociological and environmental variables should be considered in health risk analyses and control plans as these factors can affect human cases alone or in combination [11]. The potential additional variables that must be considered are (1) tsutsugamushi disease infection rates, (2) closeness to the relative proportion of female mites that pass on infections to their progeny, (3) human activities, (4) biting habits of chigger mites, (5) human populations at collection spots, and (6) pathogenicity of *Rickettsia*.

In this study, the 56 kDa gene showed six group variations in *O. tsutsugamushi* strains from different regions. We confirmed that the Kato-related strain was the most common in chigger mites in the Republic of Korea in 2020. However, Kato-related strains were only detected in restricted areas. For example, 10 Kato-related strains were only detected in the Jeongeup region in the spring season, whereas the other two Kato-related strains were detected each in the Yeongdeok and Boseong regions in the autumn season. Three Karp-related strains were detected in Hwaseong, and one Karp-related strain was found in the Yeongdeok region in autumn. Three different strains, including three Karp-related, one Shimokoshi, and one Kawasaki, were detected in the Hwaseong region. In other studies, the Boryong strain was the most predominant in the central and southern regions of the Republic of Korea, where *L. scutellare* was found [25,26]. In this study, two Boryong strains were distributed in the southern area of both the Jeongeup and Hapcheon regions, whereas one Boryong strain was found in the Yeoju region.

Overall, we surveyed a nationwide geographical and temporal distribution of chigger mite populations and performed a molecular analysis of *O. tsutsugamushi*. We identified widely distributed chigger mite species and a high degree of diversity in *O. tsutsugamushi* strains in chigger mites from different geographical regions. These results indicate that public awareness of the exposure to chigger mite vectors of *O. tsutsugamushi* during outdoor activities, especially in the autumn, is an area of interest. Additional ecological and geographical research on chigger mite vectors will improve our understanding of tsutsugamushi disease risks in the Republic of Korea. Due to globalization and climate change, the risk of invasion of vector-borne diseases via human activity has increased, and thus improved monitoring and long-term surveillance are of great interest for public health.

## Figures and Tables

**Table 1:** Geographical distribution of chigger mite species collected from sticky traps in the Republic of Korea in 2020.

Regions	No. of Collected Chigger Mites per Species																Total
	*L. scu*	*L. palp*	*N. kwa*	*N. tam*	*L. pall*	*L. tec*	*H. miy*	*L. ori*	*N. asa*	*N. jap*	*N. mit*	*N. nag*	*N. gar*	*E. kor*	*L. zet*	Un	
Yeoju	0	15	0	0	102	0	0	0	0	5	0	0	0	0	0	0	122
Paju	15	4	7	5	2	0	0	0	0	0	0	0	0	3	0	0	36
Hwaseong	380	8	0	53	115	8	3	8	0	0	18	1	0	0	0	0	594
Gangneung	0	1	156	1	16	0	1	0	0	0	0	0	0	0	0	0	175
Cheorwon	16	35	157	193	18	119	0	7	0	5	0	0	0	0	0	0	550
Okcheon	14	159	0	19	50	0	11	0	0	0	0	0	0	0	0	0	253
Boryeong	3	0	0	0	8	0	0	0	0	0	0	0	2	3	0	0	16
Yesan	10	65	0	28	30	0	24	2	0	0	0	0	0	0	1	0	160
Jeongeup	66	6	1	108	0	0	0	0	0	12	0	12	6	0	0	0	211
Jinan	11	46	73	41	1	0	8	0	27	0	0	0	0	0	0	0	207
Boseong	301	37	53	0	34	0	5	1	0	0	0	0	0	0	0	0	431
Gimcheon	0	2	0	0	18	0	1	1	0	0	0	0	0	0	0	0	22
Yeongdeok	0	146	5	0	2	0	0	0	0	1	0	0	0	1	0	3	158
Geoje	393	112	0	0	2	0	0	0	0	0	0	0	0	0	0	0	507
Hapcheon	61	78	0	0	0	0	0	0	0	0	0	0	0	0	0	0	139
Seogwipo	15	11	0	0	20	0	0	10	0	0	0	0	0	0	0	0	56
Total	1285	725	452	448	418	127	53	29	27	23	18	13	8	7	1	3	3637

*L. scu, Leptotrombidium scutellare; Leptotrombidium palp, Leptotrombidium. palpale; N. kwa, Neotrombicula kwangneungensis; N. tam, Neotrombicula tamiyai; L. pall, Leptotrombidium pallidum; L. tec, Leptotrombidium tectum; L. zet, Leptotrombidium zetum; H. miy, Helenicula miyagawai; L. ori, Leptotrombidium orientale; N. asa, Neoschoengastia asakawai; N. jap, Neoschoengastia japonica; N. mit, Neoschoengastia mitamurai; N. nag, Neoschoengastia nagayoi; N. gar, Neoschoengastia gardellai; E. kor, Euschoengastia koreansis*; Un, unidentified.

**Table 2:** Number of collected wild rodents and chigger mites from wild rodents in the Republic of Korea in 2020.

Species	No. of Collected Rodents (%)	No. of Collected Chigger Mites	Chigger Index
*Apodemus agrarius*	381 (76.4)	45,222	118.7
*Crocidura* spp.	94 (18.8)	992	10.6
*Craseomys regulus*	12 (2.4)	2314	192.8
*Micromys minutus*	9 (1.8)	1118	124.2
*Apodemus peninsulae*	2 (0.4)	283	141.5
*Microtus fortis*	1 (0.2)	224	224.0
Total	499	50,153	100.5

Chigger index, no. of collected chigger mites/no. of collected rodents.

**Table 3:** Data collection of regions and infections by *Orientia tsutsugamushi* in chigger mites from wild rodents sampled in the Republic of Korea in 2020.

Regions	No. of Collected Rodents/Installed Traps	No. of Rodents Infested with Chigger Mites (%)	No. of Collected Chigger Mites	Chigger Index	No. of Tested Chigger Mites (No. of Pools)	No. of *Ot*-Positive Chigger Mite Pools	MIR (%) of Chigger Mites
Yeoju	34/400	31 (91.2)	6623	194.8	3309 (129)	1	0.03
Paju	26/400	14 (53.8)	2099	80.7	1073 (38)	0	0
Hwaseong	29/400	26 (89.7)	4175	144.0	2086 (80)	5	0.2
Gangneung	74/400	51 (68.9)	3903	52.7	1949 (94)	0	0
Cheorwon	40/400	33 (82.5)	3357	83.9	1672 (76)	2	0.1
Cheongju	29/400	22 (75.9)	2973	102.5	1482 (63)	0	0
Boryeong	24/400	19 (79.2)	1170	48.8	582 (21)	0	0
Yesan	26/400	23 (88.5)	3458	133.0	1728 (72)	0	0
Jeongeup	29/400	24 (82.8)	3011	103.8	1506 (61)	11	0.7
Jinan	44/400	39 (88.6)	6628	150.6	3307 (134)	0	0
Boseong	26/400	22 (84.6)	2930	112.7	1463 (61)	1	0.07
Gimcheon	19/400	11 (57.9)	613	32.3	245 (14)	0	0
Yeongdeok	21/400	20 (95.2)	3126	148.9	1562 (63)	2	0.1
Geoje	24/400	18 (75.0)	1782	74.3	894 (27)	0	0
Hapcheon	24/400	18 (75.0)	3797	158.2	1897 (49)	1	0.1
Seogwipo	30/400	11 (36.7)	508	16.9	182 (16)	0	0
Total	499/6400	382 (76.6)	50,153	100.5	24,937 (998)	23	0.09

Chigger index, no. of collected chigger mites/no. of collected rodents; *Ot*, *Orientia tsutsugamushi*; MIR, minimum infection rate (no. of positive pool of mites/total no. of mites tested × 100).

## Data Availability

Data supporting the conclusions of this article are included within the article. The newly generated sequences were submitted to the GenBank database under the accession numbers MZ146353–MZ146375. The datasets used and/or analyzed during the present study are available from the corresponding author upon reasonable request.
